# Thirty-Year Trends in Anxiety Disorders Incidence Across China, Japan, and Republic of Korea: An Age–Period–Cohort Analysis Based on GBD 2021

**DOI:** 10.3390/healthcare13121376

**Published:** 2025-06-09

**Authors:** Yifan Hao, Hu Zhao, Ruhai Bai, Zhixian Xu, Yu Feng, Hui Gu

**Affiliations:** 1 School of Marxism, Nanjing University of Science & Technology, Nanjing 210094, China; haoyifan@njust.edu.cn (Y.H.);; 2 School of Public Affairs, Nanjing University of Science & Technology, Nanjing 210094, China

**Keywords:** anxiety disorders, incidence, East Asia, China, Japan, Republic of Korea, age–period–cohort model

## Abstract

**Background:** East Asia accounts for the highest number of anxiety disorders cases globally, with China, Japan, and Republic of Korea representing 17.5% of global incidence according to GBD 2021. These nations offer a unique context for examining how different modernization paths impact anxiety disorders patterns. This study examined anxiety disorders incidence trends across these countries from 1992 to 2021 to compare disease burdens and inform public health strategies. **Methods:** Using GBD 2021 data, we employed age–period–cohort analysis to evaluate factors affecting anxiety disorders incidence, examining age-specific rates, period effects, and birth cohort influences. **Results:** All three countries experienced sharp increases in anxiety disorders following COVID-19. Age-standardized incidence rates decreased by 4.89% in China and 10.52% in Japan from 1992 to 2019, while remaining stable in Republic of Korea. Net drift was −0.40% for China, −0.50% for Japan, and approximately 0 for Republic of Korea. Local drifts were positive among older adults in China and Japan, and middle-aged adults in Republic of Korea. Longitudinal age curves showed inverted V-shaped patterns, peaking in the 10–14 age group across all three countries. China and Republic of Korea exhibit a second peak during middle age, while Japan shows continuous decline after the 10–14 age group. Period and cohort effects indicating overall decreases in China and Japan, with minimal changes in Republic of Korea. **Conclusions:** Different modernization trajectories have produced distinct anxiety disorders patterns across these East Asian nations. The elevated risk among adolescents across all countries warrants targeted interventions, while high risks among middle-aged adults in China and Republic of Korea requires age-specific approaches. Countries should utilize their healthcare systems’ strengths to create targeted strategies for reducing anxiety disorders while addressing pandemic-related mental health impacts.

## 1. Introduction

Anxiety disorders represent one of the most prevalent mental health conditions globally, characterized by intense fear and distress often accompanied by physiological symptoms, resulting in substantial disease and economic burdens. Data from the Global Burden of Disease (GBD) 2021 study indicate that the number of anxiety disorders worldwide in 2021 was 53.91 million and resulted in an estimated 42.51 million Disability-Adjusted Life Years (DALYs). Of particular concern, the COVID-19 pandemic has significantly exacerbated this trend, with the COVID-19 Mental Disorders Collaborators estimating that the global number of cases of anxiety disorders increased by 76 million, or 25.6%, as a result of the pandemic, resulting in 44.5 million DALYs lost in 2020 [1]. In addition, the age-standardized DALYs share for anxiety disorders has increased the most among the 25 leading tertiary causes over the last dozen years (2010 to 2021), at 16.7 [2]. At the economic level, anxiety disorders and depression are projected to result in more than 12 billion days of lost productivity annually, with global economic losses amounting to more than USD 1 trillion and continuing to increase over time [3].

China, Japan, and Republic of Korea are all East Asian countries, and the estimated global share of anxiety disorders incidence cases in 2021 for all three countries is 17.5%, showing a 6.5% decrease from 1992 (https://vizhub.healthdata.org/gbd-results/, accessed on 12 July 2024). In terms of economic trajectory, all three countries have undergone remarkable transitions, but at different paces: Japan achieved high-income status by the late 1960s, Republic of Korea reached similar levels around 2000 and caught up with Japan by 2014, while China began its rapid-growth phase in the late 1970s and retains the highest remaining catch-up potential, with each country’s development leading the next by approximately 27 to 30 years [4]. These economic changes have directly influenced the construction and improvement of mental health service systems in each country, with Japan establishing a mature mental health service network earlier, Republic of Korea gradually improving related services after 2000 [5], and China increasing its efforts to build a mental health system in recent years [6]. This staggered development provides us with a unique perspective to study the association between socioeconomic development and anxiety disorders. Despite their different developmental trajectories, all three countries share fundamental Confucian cultural foundations that profoundly shape social structures and individual behaviors. This shared cultural heritage creates dual psychological effects: while Confucian values like harmony and collective solidarity provide mental health protection through social support systems, the emphasis on filial piety and family honor can generate significant stress and stigma around mental illness, often leading to delayed help-seeking behaviors [7]. However, the varying pace of economic modernization across these nations has created distinct challenges to traditional cultural frameworks. While maintaining core Confucian principles, each country has experienced different intensities and timing of social transformation, including evolving family structures, changing intergenerational relationships, and shifting value systems between traditional expectations and modern realities. This unique combination of shared cultural background with divergent modernization experiences provides an exceptional natural laboratory for examining how socioeconomic development influences anxiety disorders patterns within similar cultural contexts. These economic burden has been demonstrated in various countries, such as in Japan where the total cost of anxiety disorders was JPY 2.4 trillion (USD 20.5 billion) in 2008 [8], and in China where the total annual cost of mental disorders increased from USD 21 billion in 2005 to USD 88.8 billion in 2013 [9].

Several previous studies have discussed and projected trends in the burden of disease for anxiety disorders globally, nationally, or regionally [10,11,12], which provides an important reference for understanding the management of anxiety disorders globally. However, while the GBD study provides valuable epidemiological data on anxiety disorders, it lacks in-depth analysis of the complex interplay between age, period, and cohort effects within specific cultural contexts. The novelty of this study lies in employing age–period–cohort (APC) modeling to disentangle these multifaceted effects in East Asian populations. Specifically, our research provides a comprehensive cross-national comparison among China, Japan, and Republic of Korea—three countries representing distinct stages of socioeconomic development within a shared cultural framework—while examining anxiety disorders patterns against the backdrop of East Asia’s unprecedented social and economic transformation. In this study, based on the GBD 2021 database, we systematically analyzed the long-term trends in the incidence of anxiety disorders in China, Japan, and Republic of Korea between 1992 and 2021 and the potential influencing factors by using the APC model. Specifically, we aimed to (1) quantify and compare the age-standardized incidence rates of anxiety disorders across the three countries; (2) decompose the independent contributions of age, period, and birth cohort effects on anxiety disorders incidence in each nation; (3) examine whether these effects exhibit gender-specific variations both within and between countries; and (4) investigate potential correlations between the observed epidemiological trends and key socioeconomic transformations in East Asia. The results of the study will help understand the incidence characteristics of anxiety disorders in East Asia, explore the relationship between socioeconomic development and mental health, and provide empirical evidence to improve the regional mental health service system. At the policy level, the analysis of different age groups and birth cohorts in this study can inform the development of targeted intervention strategies, especially regarding resource allocation in the post-epidemic era, strengthening screening and early intervention for high-risk populations, and promoting the integration of mental health services into the primary healthcare systems. Additionally, comparative analysis of the mental health policies adopted by the three countries at different development stages and their effectiveness offers important insights for establishing regional collaborative mechanisms and developing cross-culturally adapted mental health promotion programs, ultimately reducing the burden of anxiety disorders and improving population mental health.

## 2. Materials and Methods

### 2.1. Data Source

Data for this study were obtained from the GBD 2021. The GBD 2021 database encompasses comprehensive health metrics across 371 diseases and injuries in 204 countries and territories from 1990 to 2021, including incidence, prevalence, years lived with disability (YLD), years of life lost (YLL), disability-adjusted life years (DALYs), and health-adjusted life expectancy (HALE) [2]. GBD data, obtained through comprehensive literature reviews, rigorous data screening, and sophisticated modeling processes, ensures high reliability and accuracy, making it an ideal data source for examining long-term trends in anxiety disorders incidence [13]. The data are publicly accessible through the Institute for Health Metrics and Evaluation website (https://vizhub.healthdata.org/gbd-results/) and have been de-identified, ensuring that ethical approval was not required for this study. Data access and usage complied with the guidelines and terms of use of the GBD study.

In the GBD framework, anxiety disorders are classified as non-fatal Level 3 causes, encompassing all specific anxiety types defined by both DSM and ICD criteria. Cases are identified through the following diagnostic codes: DSM-IV-TR: 300.0–300.3, 208.3, 309.21, 309.81; ICD-10: F40–42, F43.0, F43.1, F93.0–93.2, F93.8. The GBD 2021 anxiety disorders data incorporate multiple versions of diagnostic criteria, including DSM (DSM-III, DSM-III-R, DSM-IV, DSM-IV-TR, DSM-5, and DSM-5-TR) and ICD (ICD-9, ICD-10, and ICD-11). Notably, anxiety disorders caused by general medical conditions or substance use are excluded from these estimates. The data of anxiety disorders in GBD 2021 were extracted through three age-sex segmentation processes: first, estimates were further split by sex and age based on the available data; second, the remaining male and female estimates in the dataset were split using a meta-regression Bayesian-regularized trimming (MR-BRT) analysis; and third, for studies that reported prevalence estimates for the 25-and-olderage group, the prevalence-age model estimated using DisMod-MR 2.1 was used to split these into consecutive 5-year age groups, as accessed through the Institute for Health Metrics and Evaluation website (https://ghdx.healthdata.org/gbd-2021/, accessed on 12 July 2024).

### 2.2. Data Analysis

Incidence is defined as the number of new cases of a disease occurring in a specified population during a given time period, expressed as a rate per population. For this analysis, we utilized age-standardized incidence rates (ASIRs) of anxiety disorders per 100,000 population, standardized using the GBD 2021 global reference population to enable cross-country and temporal comparisons. The GBD modeling process generates point estimates (means of 1000 Monte Carlo draws) and 95% uncertainty intervals (UIs, 2.5th–97.5th percentiles) that capture uncertainty from data sampling, model specification, and parameter estimation. This study employed the APC model to analyze anxiety disorders incidence data from China, Japan, and Republic of Korea, estimating the effects of age, period, and cohort factors on incidence rates in each country. The model examines temporal patterns of anxiety disorders across different dimensions: age effects reflect changes in incidence rates attributable to biological and social developmental processes; period effects capture risk variations across different time points; and cohort effects demonstrate overall risk changes across different birth cohorts. To evaluate these three effects comprehensively, we estimated five key parameters [14]. Net drift is calculated as the overall log-linear trend across calendar periods and birth cohorts, indicating the overall annual percentage change in age-adjusted rates throughout the study period. Local drift is computed as age-specific log-linear trends across calendar periods and birth cohorts for each age group, quantifying the age-specific annual percentage changes derived from the slope of the cohort rate ratio curve. The longitudinal age curve represents the age effect, i.e., the expected age-specific incidence after adjusting for period and cohort effects. Period and cohort rate ratios quantify period and cohort effects, respectively, and expressed the relative risks in each period or cohort compared to their reference counterparts.

To organize the data for APC analysis, we divided the observation cohort into consistent 5-year intervals. The study population (ages 5–84) was segmented into 16 age groups, while the observation period (1992–2021) was divided into 6 time periods. This approach yielded 21 birth cohort groups spanning from 1910 to 2010. We designated the 40–44 age group, 2002–2006 period, and 1962–1966 birth cohort group as references for comparative analysis. Parameter estimation was conducted using the National Cancer Institute’s APC Web Tool (https://analysistools.cancer.gov/apc/, accessed on 15 July 2024), which employs an intrinsic estimating function approach to address the identification problem inherent in APC modeling. This method assumes smooth effect transitions between adjacent birth cohorts, presupposes no interaction among age, period, and cohort effects, and applies a log-linear model that assumes incidence rates follow a Poisson distribution. The median values within each age group, period group, and cohort group served as reference points for estimation calculations. Our study primarily focused on categorical comparisons of anxiety disorders incidence across countries, time periods, and demographic characteristics. Given that anxiety disorders incidence data in large populations typically exhibit skewed distributions, we selected chi-square tests as the most appropriate statistical method for analyzing these categorical differences. Results yielding *p*-values less than 0.05 were considered statistically significant. The main analysis and graphing in this study were implemented using R program version 4.3.0 (https://www.R-project.org/, accessed on 23 October 2024).

## 3. Results

### 3.1. Trends in Anxiety Disorders Incidence in China, Japan, and Republic of Korea from 1992 to 2021

**Figure 1:**
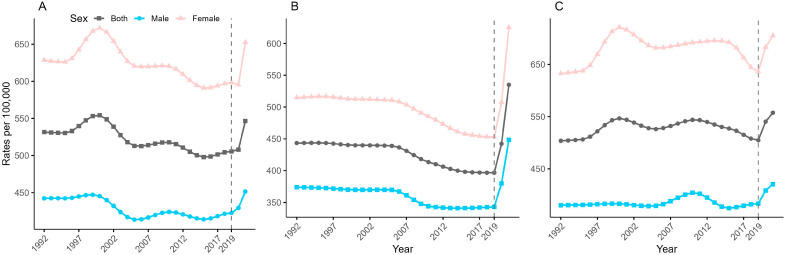
Trends in the age-standardized incidence rates (ASIRs) of anxiety disorders in China (**A**), Japan (**B**), and Republic of Korea (**C**) from 1992 to 2021.

Figure 1 shows the trends in age-standardized incidence rates by sex of anxiety disorders in China, Japan, and Republic of Korea from 1992 to 2021, using the global age-standardized population of GBD 2021. The results show that the incidence of anxiety disorders has increased sharply in all three countries after COVID-19 and has been consistently higher in females than in males. Prior to the pandemic, the overall age-standardized anxiety disorders incidence in China and Japan had reduced, with age-standardized incidence rates in 2019 decreasing by 4.89% and 10.52%, respectively, compared with 1992, and the overall age-standardized anxiety disorders incidence in Republic of Korea returned to its initial level after more than two decades of change. The COVID-19 pandemic significantly affected the anxiety disorders incidence. From 2019 to 2021, the rates increased in China—from 505.68 cases (95% CI: 430.76–592.50) to 546.51 cases (95% CI: 454.19–662.14), representing an 8.07% increase; in Japan—from 396.76 cases (95% CI: 332.20–474.27) to 535.01 cases (95% CI: 446.80–638.38), a 34.84% increase; and in Republic of Korea—from 504.82 cases (95% CI: 410.13–634.85) to 557.56 cases (95% CI: 390.36–779.64), a 10.45% increase.

### 3.2. Local Drift and Net Drift in the Incidence of Anxiety Disorders in China, Japan and Republic of Korea

**Figure 2:**
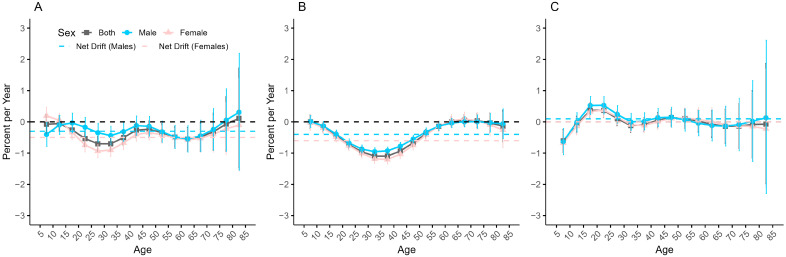
Local drift and net drift in the incidence of anxiety disorders in China (**A**), Japan (**B**), and Republic of Korea (**C**) with confidence intervals.

Figure 2 shows the overall annual change in the incidence by sex of anxiety disorders in China, Japan, and Republic of Korea, as well as the annual change in each age group. The results show that the overall net drift in the anxiety disorders incidence was −0.40% (95% CI: −0.55% to −0.25%) for China, −0.30% (95% CI: −0.45% to −0.10%) for males, and −0.50% (95% CI: −0.64% to −0.36%) for females, suggesting an overall annual decline of 0.40%, with a significant decline in females relative to males. The overall net drift in the anxiety disorders incidence in Japan is −0.50% (95% CI: −0.58% to −0.44%), with a net drift of −0.40% (95% CI: −0.51% to −0.38%) for males, and −0.60% (95% CI: −0.64% to −0.48%) for females, indicating an overall decrease of 0.50% per year, with a more pronounced decrease for females. The overall net drift in the anxiety disorders incidence in Republic of Korea is 0 (95% CI: −0.16% to 0.17%), with a net drift of 0.1% (95%CI: −0.13% to 0.26%) for males and 0 (95% CI: −0.16% to 0.18%) for females, suggesting little overall change from year to year.

The overall trend of local drift with age in the anxiety disorders incidence is similar for both males and females in all three countries, with sex differences only appearing in some age groups. In China, local drift is below 0 for both males and females in the 15–75 age group, but above 0 for males in the 75–85 age group and for females in the 5–15 age group, suggesting an increase in anxiety disorders in older males and younger females. Japanese males in the 65–75 age group and Japanese females in the 60–75 age group has local drifts higher than 0 (*p* < 0.05), while the rest of the age groups has local drifts lower than 0, which suggests an increase in the anxiety disorders incidence in the elderly. In Republic of Korea, the local drift is greater than 0 in the age groups of 15–30 years, 35–55 years, 75–84 years for males, and greater than 0 in the age groups of 15–30 years, 40–60 years for females, and less than 0 in the rest of the age groups for both males and females, which suggests that the burden of disease for anxiety disorders has shifted mainly to middle-aged people.

### 3.3. Longitudinal Age Change Curves for Anxiety Disorders Incidence in China, Japan, and Republic of Korea

**Figure 3:**
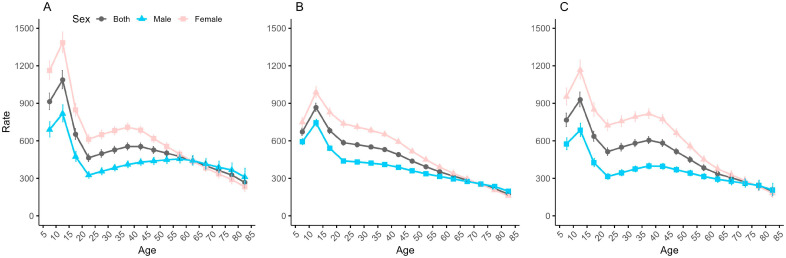
Longitudinal age-specific curves and their respective 95% confidence intervals for the incidence of anxiety disorders in China (**A**), Japan (**B**), and Republic of Korea (**C**).

Figure 3 shows the age effect, i.e., the period-adjusted trend by sex of change with age in the same birth cohort, for the incidence of anxiety disorders in China, Japan, and Republic of Korea. The results show that the anxiety disorders incidence in China, Japan, and Republic of Korea all show a trend of increasing and then decreasing, with the difference being in the rate of decline and trend of change after reaching the peak. In all three countries, the anxiety disorders incidence peaks in the age group of 10–14 years and then begins to decline. In Japan, the anxiety disorders incidence continues to decline with increasing age after the 10–14 age group. China and Republic of Korea show similar trends, with a rapid decline from the 10–14 age group to the 20–24 age group, then rising to the 35–39 age group, and declining continuously thereafter. In all three countries, the age trends in the anxiety disorders incidence in males and females are similar, but the decline in females is significantly higher than that in males in the 40–84 age group, leading to a reversal of the anxiety disorders incidence in older males over females in the three countries, whereas prior to this, the incidence in females is almost twice that of males in all age groups from 5 to 9 years old to 40–44 years old in all three countries.

### 3.4. Period-Relative Risk of Anxiety Disorders Incidence in China, Japan, and Republic of Korea

**Figure 4:**
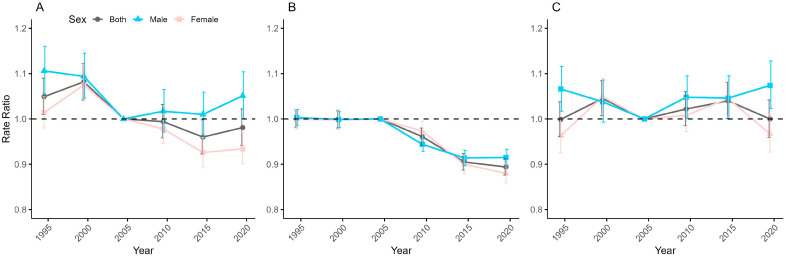
Period-relative rates and 95% confidence intervals for the incidence of anxiety disorders in China (**A**), Japan (**B**), and Republic of Korea (**C**).

Figure 4 shows the period effects by sex of anxiety disorders incidence in China, Japan, and Republic of Korea, i.e., the trends in period-relative rates after adjusting for age and nonlinear cohort effects. The results show that the period effects of anxiety disorders incidence rates vary widely among the three countries. In China, the period ratio of anxiety disorders incidence by sex generally shows an increasing and then decreasing trend, with an increase from 1992–1996 to 1997–2001, and a gradual decrease from 2002–2006 to 2012–2016, followed by an increasing trend. The period proportions of anxiety disorders incidence by both sex in Japan basically remain flat and then gradually decline, remaining almost unchanged from 1992–1996 to 1997–2001, and then starting to decline continuously. The period proportions of anxiety disorders incidence by both sex in Republic of Korea show an M-shaped change, with an upward trend from 1992–1996 to 1997–2001 and from 2002–2006 to 2007–2011, and a downward trend from 1997–2001 to 2002–2006 and from 2012–2016 to 2017–2021. Of the three countries, only Japan’s period effects for males and females are relatively similar, while the sex differences in period effects are more pronounced in China and Republic of Korea. However, in both latter countries, male period effects exhibit similar patterns, characterized by an initial decrease followed by a subsequent increase, with relative risk values consistently remaining above 1.0. In contrast, the period effects for females show significant differences between these countries.

### 3.5. Cohort-Relative Risk of Anxiety Disorders Incidence in China, Japan, and Republic of Korea

**Figure 5:**
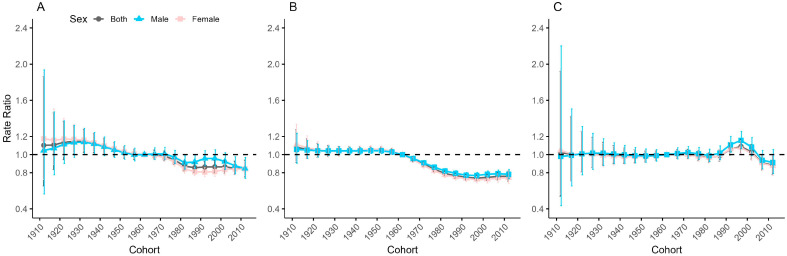
Cohort-relative rates and 95% confidence intervals for the incidence of anxiety disorders in China (**A**), Japan (**B**), and Republic of Korea (**C**).

Figure 5 shows the cohort effects by sex of anxiety disorders incidence in China, Japan, and Republic of Korea, i.e., the trends of cohort-relative rates after adjustment for age and nonlinear period effects. The results show that the cohort effects of anxiety disorders incidence in the three countries are significantly different. In China, the cohort-relative ratio of anxiety disorders incidence by both sex increase slowly from the 1912–1916 cohort to the 1927–1931 cohort and then decline continuously to the 1987–1991 cohort, followed by small changes in the cohort-relative ratio. The cohort-relative ratio of anxiety disorders incidence by both sexes in Japan remains stable from the 1912–1916 cohort to the 1952–1956 cohort, decline continuously from the 1952–1956 cohort to the 1992–1996 cohort, and then increase slightly in recent years as a result of the cohort effect. The cohort-relative ratio of anxiety disorders incidence by both sex in Republic of Korea remain relatively flat from the 1912–1916 cohort to the 1977–1981 cohort, increase from the 1982–1986 cohort to the 1997–2001 cohort, and decline thereafter. Cohort effects for anxiety disorders incidence are generally larger for men than for women in all three countries, and trends in cohort effects are similar for men and women in both Japan and Republic of Korea. The sex differences in the cohort effects of anxiety disorders incidence are larger in China, with the cohort risk increasing first and then decreasing for males born after 1977–1981, in the opposite direction of the change for females and with a larger magnitude of change than for females.

## 4. Discussion

To the best of our knowledge, this is the first study based on the GBD 2021 to analyze and compare trends in the anxiety disorders incidence in three East Asian countries using the APC model. Our APC analysis, which decomposed temporal trends into age, period, and cohort effects using predefined 5-year intervals, revealed that the age-standardized incidence of anxiety disorders in China, Japan, and Republic of Korea increased significantly after the COVID-19 pandemic, and the incidence was consistently higher in females than in males. Prior to the COVID-19 pandemic, i.e., from 1992 to 2019, the incidence trends in the three East Asian countries varied over time, with a continuing decline in Japan, three increases and two decreases in China, and two increases and two decreases in Republic of Korea. The calculated local drift analysis revealed age-specific variations in these patterns across all three countries. There were similar age effects for anxiety disorders incidence trends in the three countries, with significant differences in period and cohort effects. The morbidity trends observed above are the result of a combination of age, period, and cohort effects that arise from the combined effects of collective culture, social change, major events, and individual life course.

After the pandemic, a sharp increase in anxiety disorders incidence appeared in all three countries regardless of sex. Daily infection rates, decreasing mobility, and daily excess mortality rates are notable indicators for observing the impact of the COVID-19 pandemic [1]. Declining human activity leads to social isolation, reduces emotional support, creates economic and lifestyle uncertainty, exacerbates economic stress, and reflects overlapping individual behaviors and policies. Rising estimated daily infection rates increase concerns about individual and household exposure to the virus and its consequences, heighten public concern about potential healthcare shortages, and cause economic and social instability. Increasing mortality rates have raised awareness of the seriousness of COVID-19, triggering fear, helplessness, and heightened anxiety, and bringing collective grief and stress. Scholars in the three countries designed specific studies to discover the specific mechanisms by which COVID-19 affects anxiety disorders. Chinese scholars found that regardless of geographic location, parents who experienced quarantine reported higher levels of GAD symptoms [15]. A Japanese survey reported the economic impact of the epidemic on the severity of anxiety disorders [16]. In addition to reporting these conventional influences, Republic of Korea scholars found that changes in sleep patterns due to COVID-19 and post-infection health concerns were major contributors to anxiety disorders [17]. In fact, lockdowns that cause this immobility have both advantages and disadvantages; by decreasing the rate of infection, the lockdown may reduce the incidence of anxiety disorders [18]. And based on the pathways of anxiety disorders impact during and after the pandemic, COVID-19 will change mental health in the coming decades, a prediction that is initially supported in GBD 2021 by the rapid growth trends and high incidence rates after 2019 in the individual countries.

Our age-standardized incidence rate analysis consistently showed that anxiety disorders incidence was consistently higher in women than in men in all three countries, and in most age groups women suffered from anxiety disorders at twice the rate of men, which is consistent with findings in epidemiology. There are two main explanations for this, a neurobiological one and a psychosocial one. For the former, it has been concluded that anxiety disorders are influenced by a combination of genetic, brain structure, hormonal, and neurobiological factors [19,20]. Studies have shown that sex differences in stress-related receptors in the brain such as the hippocampus, amygdala, and prefrontal cortex make females more susceptible to stress response dysregulation, which can lead to mood and anxiety disorders [21]; for example, reduced concentrations of glycerophosphorylcholine (GPC + PC) and glutamate (Glu) in males with anxiety disorders demonstrate that these two brain metabolite concentration changes play a role in the anxiety spectrum [22]. However, only a relatively small number of studies (2%) of sex differences in anxiety disorders have focused on the female brain, and Kelimer’s research suggests that sex hormones (especially estrogen) may have a direct effect on the molecular mechanisms mediating synaptic plasticity in the hippocampus and forebrain cortex during fear extinction [23]. Indeed, in addition to effects within brain structures, the role of sex hormones on anxiety involves biological, behavioral, and cognitive processes, which are intricately linked, with rises in estradiol and luteinizing hormone acting both to protect and increase vulnerability [24]. Hormonal fluctuations also play an important role in how other biological factors (tryptophan, serotonin, etc.) interact with sex hormone production. Another explanation for anxiety disorders is psychosocial factors, i.e., women are oppressed by socio-cultural as well as structural factors such as sex-based violence, are more susceptible to emotional stress than men, are more likely to experience trauma related to domestic violence or sexual abuse, and are at significantly increased risk for anxiety disorders. Finally, sex differences in the anxiety disorders incidence are also explained by the expression of emotional distress due to social norms; in general, men and older adults with anxiety disorders are very unlikely to seek help, and men are less likely to attend routine medical appointments [20].

In the trends of anxiety disorders incidence in the three East Asian countries, a partially similar age effect is observed, i.e., anxiety disorders incidence peaks at ages 10–14 years and then begins to decline to varying degrees. This reflects the high incidence of mental health problems in younger populations and is also related to adolescents’ heightened sensitivity to social pressures and tension between academic and familial expectations, especially in East Asian countries, where failure to meet societal expectations often leads to anxiety. Physiological and developmental factors are also the main reasons why anxiety disorders peak at this age, as 10–14 years of age is an important neurodevelopmental stage, especially when brain regions associated with emotion regulation such as the prefrontal cortex and the limbic system are still not fully mature, which will lead to limitations in an individual’s ability to regulate their emotions [25]. In addition, the hormonal changes, especially the increase in sex hormones, can cause mood swings that make adolescents vulnerable to anxiety disorders. Adolescents also tend to face a sudden increase in academic pressures as well as the complexity of peer pressures at the age of 10–14 years [26], and in China, Japan, and Republic of Korea, adolescents in this age group usually face pressures to advance to higher education. Additionally, conflict or rejection in peer relationships may also lead to psychological stress. However, among the three countries, only Japan’s trend of anxiety disorders burden has been decreasing with age, while China and Republic of Korea only decline to the 20–24 age group after reaching the peak incidence at 10–14 years old, followed by a secondary rise peaking at ages 35–39 before the burden continues to decrease. This trend in China and Republic of Korea is similar to other studies that have concluded that anxiety disorders usually peak in middle age and then decrease with age, showing a chronic course that does not persist into old age in most cases [10]. This pattern reflects the combination of multiple stressors that characterize midlife: intense professional competition, heavy family responsibilities, increased financial pressures, and the onset of declining physical health. However, the pattern of declining anxiety disorders incidence after puberty in Japan should not be interpreted as a genuine improvement in mental health but rather as a cultural reporting bias exacerbated by age. Such an explanation is particularly important in light of Japan’s paradoxically high suicide rate, which has often been associated with higher levels of mental disorders in previous findings [27]. Japanese culture promotes stigmatizing attitudes that attribute mental illness to “personality weakness”, while the cultural phenomenon of “honne/tatemae” (authentic feelings versus publicly expressed opinions) discourages genuine symptom disclosure. This cultural suppression becomes increasingly pronounced in adulthood, as evidenced by approximately 70% of individuals with mental disorders in Japan not receiving treatment [28]. Research demonstrates that nearly one-quarter of Japanese respondents recommended “deflection” strategies when confronted with mental health scenarios, reflecting cultural preferences for maintaining social harmony without disclosing personal struggles [29]. This cultural context explains why anxiety disorders incidence appears to decline with age in Japan while suicide rates remain exceptionally high, indicating that psychological distress persists but becomes progressively concealed through cultural mechanisms rather than authentic recovery.

The anxiety disorders incidence in the three East Asian countries was significantly affected by the period effect. In China and Japan, the anxiety disorders incidence declined significantly after 2000 and remained at a low level, while the period effect began to decline in both countries and has been less than 1 since then, suggesting that the decline in the anxiety disorders incidence during this period has a strong affinity with the period effect. Economic development can explain part of the period effect. China’s economic reform and Japan’s economic recovery in recent decades have been a great success, especially since China surpassed Japan to become the second largest economy after the United States after 2002, and the economic boom has reduced poverty [30], alleviating many stressors associated with anxiety disorders such as economic insecurity and unemployment, and technological advances brought about by the economic development have made it easier for anxiety disorders to be recognized and managed, and mental health has continued to improve. However, after a certain period of economic development comes the challenge of inequality [31], as evidenced by the period effect, which is on the rise in both countries after 2010. More likely to explain the period effect are the efforts at public health and mental health reform in both countries. After 2000 the Chinese government began to take more effective measures to fund public health as well as psychiatric services, including ensuring that most psychotropic medications were included in basic medical insurance in 2005, as well as a plan to set up 550 psychiatric hospitals and psychiatric units within general hospitals. Additionally, the government successively launched and implemented the New Rural Cooperative Medical Scheme (NRCMS) in the early 2000s, the National Community Service Model (Project 686) in 2004, and the National Mental Health Plan (2015–2020); the enactment and implementation of national and local mental health laws have safeguarded the rights of people with mental disorders and reduced the risk of anxiety disorders morbidity [32,33,34]. Japan has also taken a number of policy actions in the field of mental health to strengthen the early diagnosis and treatment of anxiety disorders, with the core strategy being the National Health Promotion Plan, a policy on NCDs that was first formally introduced in 1978 and revised every 10 years, with the third and fourth editions known as Health Japan 21 (2000–2012) and Health Japan (2013–2022), respectively. These plans address Japan’s rapidly aging social changes and emphasize primary prevention of NCDs, recommending targets in key risk factor areas [35]. More differently, the time trend and period effect of the age-standardized incidence of anxiety disorders in Republic of Korea during the observation period showed an M-shape and peaked around 2000 and 2015, implying the presence of risk factors that kept anxiety disorders on the rise during the observation period. Although modernization and public health policies such as the enactment of the Mental Health Act in 1995 have brought some positive changes to the mental health system in Republic of Korea, the overall mental health status in Republic of Korea remains low and suicide rates have not improved significantly, which is significantly related to the problems in its mental health service system [36]. Specifically, the low percentage of people with mental illnesses receiving health services, the low budget for mental health services in Republic of Korea, and the lack of organizations specializing in mental health research and development in Republic of Korea [5] may contribute to the high anxiety disorders incidence. Finally, in most developed countries where mental illness is the largest burden of disease, an increase in the use of mental health services is likely to lead to a rise in the number of patients, and an increase in the use of mental health services alone will not lead to a decrease in the incidence of common mental disorders (CMDs) [37]. Periodic factors such as changes in economic conditions and large-scale disasters also influence the incidence of anxiety disorders and mental health service use, for example, the severe economic inequality in the society of Republic of Korea, especially the Asian financial crisis in 1997, which caused a surge in unemployment and a rise in household indebtedness in Republic of Korea, may be related to the higher period effect of the 2000s.

The cohort effects of anxiety disorders incidence similarly differed across the three countries. For China and Japan, the anxiety disorders incidence in the post-1960 birth cohort was lower, and the cohort effect declined significantly faster in Japan than in China. In addition to the sustained efforts in public health in both countries, increased social stability due to economic development, political relations, and changes in international status may be the influencing factors for the significant cohort effect. After the founding of the People’s Republic of China (PRC), China has accumulated a wealth of experience by going through a primary phase centered on prevention, a transformation phase favoring treatment, a recovery phase after SARS, and a new phase of an equitable and people-centered system [38]; Japan’s Mental Health Law was enacted in 1950 after World War II, which facilitated the construction of governmental psychiatric hospitals, and then in the following decades a variety of social events and governmental adjustments led to the enactment of successive mental health laws regulating the care of people with mental disorders [39]. For both countries, the development of the mental health system and related laws was closely related to social stability. After the founding of the PRC in 1949, psychiatric hospitals were established primarily to maintain social security and stability. The community mental health initiative, which began in the late 1950s, aimed at preventing and treating psychiatric disorders while ensuring that people with mental disorders did not disrupt the social order. Not only did the government view mental health as a public health issue but also as an integral part of social cohesion [6]. Therefore, the cohort effect brought about by public health improvements could only be maintained for a certain period of time, and two decades later, the rapid socioeconomic changes in both countries—such as urbanization, industrialization, and globalization—again led to a serious increase in the incidence of mental disorders, including anxiety disorder. As a result, the two countries faced new mental health challenges, and the cohort effect remained at the same level and has not changed significantly in recent decades. In summary, social stability significantly influences mental health outcomes in China and Japan through cultural attitudes, economic conditions, and government initiatives [29,40]. Republic of Korea is different, with a more pronounced cohort effect not appearing until after 1990, with a gradual increase in the degree of cohort influence for those born between 1990 and 2000, and a gradual decrease in the degree of cohort influence for those born between 2000 and 2010. The 1990–2000 born cohort faced widespread family unemployment and economic instability due to dramatic economic changes, especially the financial crisis of 1997, and the persistently rising suicide rate had a more severe psychological impact on the cohort at that stage [41]. Additionally, despite the Mental Health Act passed in 1995, which expanded the number of national mental health centers and related facilities, the effectiveness of psychiatric treatment has been less than optimal, which is closely related to the more heavily Confucian culture in the Republic of Korea. After 2000, the Republic of Korea’s government began to pay more attention to mental health issues by launching several mental health intervention programs, such as the first National Suicide Prevention Initiative (NSPI) implemented in 2004, and this generation has benefited from the change in policy and the shift in attitudes towards mental health issues in Republic of Korea’s society.

There are several limitations of this study that need to be considered. First, a primary limitation is the reliance on modeled data from the GBD 2021 rather than direct epidemiological measurements. GBD estimates are derived from statistical modeling approaches that integrate data from multiple sources to generate comprehensive estimates where direct measurements may be incomplete or unavailable [2]. This modeling approach introduces inherent uncertainties that vary by region and time period, potentially affecting the accuracy of trend analyses. Second, the GBD 2021 data may partially reflect differences between the three countries’ reporting systems, such as subtle variations in diagnostic criteria, regional differences in healthcare accessibility, and the potential influence of cultural factors on healthcare-seeking behaviors. However, we mitigated the effects of demographic differences by using standardized age-adjusted rates for comparison. Third, differences in data collection methods across the three countries may still introduce some degree of variation, although the GBD model corrects for these systematic differences using Bayesian regression [2]. Fourth, the present study used aggregate data with the APC model, which is subject to the ecological fallacy that interpretations at the population level may not apply to individual cases, and future studies at the individual level will be needed to corroborate the relevant findings of the present study. Finally, anxiety disorders encompass a wide range of subtypes, and future research could examine specific anxiety disorder subtypes rather than treating them as a single category to further explore changes in patterns of specific subtypes.

Based on our findings, we propose targeted policy recommendations to address the burden of anxiety disorders across these East Asian nations. For China, given the pronounced increase in anxiety disorders among young and middle-aged populations, policy interventions should prioritize workplace mental health initiatives, encompassing mandatory stress management programs, regulated working hours, and enhanced employee assistance schemes. Concurrently, strengthening primary healthcare infrastructure is essential to facilitate early detection and intervention, particularly in rapidly urbanizing areas experiencing heightened socioeconomic pressures. Japan’s policy framework should address the paradox between declining age-adjusted anxiety burden and persistently elevated suicide rates through comprehensive youth-focused suicide prevention strategies, stigma reduction via targeted public education campaigns, and expansion of culturally responsive mental health services. Critical workplace reforms addressing endemic overwork culture remain imperative. For Republic of Korea, where anxiety disorders exhibit a distinctive bimodal age distribution spanning adolescence and middle age, educational institutions require enhanced mental health screening and support infrastructures, while labor policies must emphasize workload regulation and work–life balance optimization. Cross-nationally, all three countries would benefit from systematic integration of mental health services within primary care frameworks, enhanced mental health literacy through public education initiatives, stigma reduction surrounding help-seeking behaviors, and expanded insurance coverage for psychotherapeutic interventions. Digital mental health platforms represent promising avenues for reaching underserved populations, provided they incorporate culturally sensitive design principles appropriate for East Asian contexts.

## 5. Conclusions

In general, prior to the pandemic, the incidence of anxiety disorders declined from 1992 in China and Japan, while it did not change significantly in Republic of Korea. These patterns reflect the complex interplay between shared Confucian cultural foundations and divergent modernization trajectories across the three countries. During this period, the anxiety disorders incidence continued to decline in Japan and alternated between increasing and decreasing in China and Republic of Korea. However, the apparent decline, particularly in Japan, may reflect cultural reporting biases rather than genuine improvement, as Confucian emphasis on social harmony and stigma around mental illness leads to systematic underreporting of psychological symptoms. Women and adolescents are at a higher risk of anxiety disorders, and targeted interventions, likely reflecting tensions between traditional Confucian role expectations and modern social realities, such as mental health education and knowledge dissemination, and increased attention to vulnerable groups, should be implemented for these groups. Middle-aged adults in China and Republic of Korea are also at higher risk of anxiety disorders, highlighting the importance of tailoring mental health strategies to country-specific and age-specific factors that consider how rapid economic modernization creates psychological stress as individuals navigate between traditional family obligations and contemporary socioeconomic pressures.

During the pandemic, we observed a significant increase in the anxiety disorders incidence in all three countries, but the magnitude and pattern of impact varied. These differential impacts underscore how cultural frameworks shape crisis responses, with each country’s approach reflecting distinct interpretations of Confucian values in modern governance. For example, stricter lockdown measures in China and other differences may have led to different mental health outcomes. Future studies should delve deeper into the ongoing impact of the COVID-19 epidemic on long-term mental health trends in East Asia, especially on the adaptation process of identified high-risk populations in the post-epidemic era. Meanwhile, with the development of digital health technologies, we should explore how to integrate emerging technologies such as artificial intelligence, mobile health apps, and telemedicine into anxiety screening. Such intervention may provide innovative pathways to overcome existing healthcare resource inequalities and cultural barriers. Effective interventions in East Asia must leverage Confucian strengths—such as family-centered support systems—while addressing cultural barriers to help-seeking. In addition, establishing a more standardized cross-national mental health surveillance system that accounts for cultural reporting biases would help reduce the reporting system discrepancies identified in this study. By leveraging each country’s healthcare strengths and integrating new technologies, more effective and targeted approaches can be developed to reduce the burden of anxiety disorders and improve the mental health of diverse populations.

## Data Availability

The data that support the findings of this study are publicly available from the Global Burden of Disease Study 2021 (GBD 2021) at http://ghdx.healthdata.org/gbd-results-tool, accessed on 12 July 2024. Analysis code is available from the corresponding author upon reasonable request.

## Abbreviations

The following abbreviations are used in this manuscript:

GBD	Global Burden of Disease
DALYs	Disability-Adjusted Life Years
APC	Age–Period–Cohort
ASIR	Age-Standardized Incidence Rate

## References

[1] COVID-19 Mental Disorders Collaborators (2021). Global Prevalence and Burden of Depressive and Anxiety Disorders in 204 Countries and Territories in 2020 Due to the COVID-19 Pandemic. Lancet.

[2] GBD 2021 Diseases and Injuries Collaborators (2024). Global incidence, prevalence, years lived with disability (YLDs), disability-adjusted life years (DALYs), and healthy life expectancy (HALE) for 371 diseases and injuries in 204 countries and territories and 811 subnational locations, 1990–2021: A systematic analysis for the global burden of disease study 2021. Lancet.

[3] Chisholm D., Sweeny K., Sheehan P., Rasmussen B., Smit F., Cuijpers P., Saxena S. (2016). Scaling-up Treatment of Depression and Anxiety: A Global Return on Investment Analysis. Lancet Psychiatry.

[4] Murach M., Wagner H., Kim J., Park D. (2022). Trajectories to High Income: Comparing the Growth Dynamics in China, South Korea, and Japan with Cointegrated VAR Models. Struct. Change Econ. Dyn..

[5] Roh S., Lee S.-U., Soh M., Ryu V., Kim H., Jang J.W., Lim H.Y., Jeon M., Park J.-I., Choi S. (2016). Mental health services and R&D in South Korea. Int. J. Ment. Health Syst..

[6] Liu J., Ma H., He Y., Xie B., Xu Y., Tang H., Li M., Hao W., Wang X., Zhang M. (2011). Mental Health System in China: History, Recent Service Reform and Future Challenges. World Psychiatry.

[7] Badanta B., González-Cano-Caballero M., Suárez-Reina P., Lucchetti G., De Diego-Cordero R. (2022). How Does Confucianism Influence Health Behaviors, Health Outcomes and Medical Decisions? A Scoping Review. J. Relig. Health.

[8] Sado M., Takechi S., Inagaki A., Fujisawa D., Koreki A., Mimura M., Yoshimura K. (2013). Cost of Anxiety Disorders in Japan in 2008: A Prevalence-Based Approach. BMC Psychiatry.

[9] Xu J., Wang J., Qiu C. (2016). The economic burden of mental disorders in China, 2005–2013: Implications for health policy. BMC Psychiatry.

[10] Javaid S.F., Hashim I.J., Hashim M.J., Stip E., Samad M.A., Ahbabi A.A. (2023). Epidemiology of Anxiety Disorders: Global Burden and Sociodemographic Associations. Middle East Curr. Psychiatry.

[11] Liu D., Luo M., Huang Y., Tan Y., Cheng F., Wu Y. (2024). Time trends in anxiety disorders incidence across the BRICS: An age-period-cohort analysis for the GBD 2021. Front. Public Health.

[12] Zhi Z., Yan S., Yijuan H., Jiahuan Z., Xiaohan J., Dandan C. (2024). Trends in the Disease Burden of Anxiety Disorders in Middle-Aged and Older Adults in China. BMC Psychol..

[13] Ward Z.J., Goldie S.J. (2024). Global Burden of Disease Study 2021 Estimates: Implications for Health Policy and Research. Lancet.

[14] Rosenberg P.S., Check D.P., Anderson W.F. (2014). A Web Tool for Age–Period–Cohort Analysis of Cancer Incidence and Mortality Rates. Cancer Epidemiol. Biomark. Prev..

[15] Wu S., Yao M., Deng C., Marsiglia F.F., Duan W. (2021). Social Isolation and Anxiety Disorder during the COVID-19 Pandemic and Lockdown in China. J. Affect. Disord..

[16] Ueda M., Stickley A., Sueki H., Matsubayashi T. (2020). Mental Health Status of the General Population in Japan during the COVID-19 Pandemic. Psychiatry Clin. Neurosci..

[17] Kim D.M., Bang Y.R., Kim J.H., Park J.H. (2021). The Prevalence of Depression, Anxiety and Associated Factors among the General Public during COVID-19 Pandemic: A Cross-Sectional Study in Korea. J. Korean Med. Sci..

[18] Lokman J.C., Bockting C.L. (2022). Pathways to depressive and anxiety disorders during and after the COVID-19 pandemic. Lancet Psychiatry.

[19] Jalnapurkar I., Allen M., Pigott T. (2018). Sex Differences in Anxiety Disorders: A Review. Psychiatr. Danub..

[20] Farhane-Medina N.Z., Luque B., Tabernero C., Castillo-Mayén R. (2022). Factors Associated with Gender and Sex Differences in Anxiety Prevalence and Comorbidity: A Systematic Review. Sci. Prog..

[21] Bangasser D.A. (2013). Sex Differences in Stress-Related Receptors: “Micro” Differences with “Macro” Implications for Mood and Anxiety Disorders. Biol. Sex Differ..

[22] Pigoni A., Delvecchio G., Squarcina L., Bonivento C., Girardi P. (2020). Sex differences in brain metabolites in anxiety and mood disorders. Psychiatry Res. Neuroimaging.

[23] Lebron-Milad K., Milad M.R. (2012). Sex differences, gonadal hormones and the fear extinction network: Implications for anxiety disorders. Biol. Mood Anxiety Disord..

[24] Li S.H., Graham B.M. (2017). Why are women so vulnerable to anxiety, trauma-related and stress-related disorders? The potential role of sex hormones. Lancet Psychiatry.

[25] Blakemore S.-J. (2012). Imaging Brain Development: The Adolescent Brain. NeuroImage.

[26] Kessler R.C., Berglund P., Demler O., Jin R., Merikangas K.R., Walters E.E. (2005). Lifetime Prevalence and Age-of-Onset Distributions of DSM-IV Disorders in the National Comorbidity Survey Replication. Arch. Gen. Psychiatry.

[27] Hirokawa S., Kawakami N., Matsumoto T., Inagaki A., Eguchi N., Tsuchiya M., Katsumata Y., Akazawa M., Kameyama A., Tachimori H. (2012). Mental Disorders and Suicide in Japan: A Nation-Wide Psychological Autopsy Case–Control Study. J. Affect. Disord..

[28] Ando S., Yamaguchi S., Aoki Y., Thornicroft G. (2013). Review of Mental-health-related Stigma in Japan. Psychiatry Clin. Neurosci..

[29] Kikuzawa S., Pescosolido B., Kasahara-Kiritani M., Matoba T., Yamaki C., Sugiyama K. (2019). Mental Health Care and the Cultural Toolboxes of the Present-Day Japanese Population: Examining Suggested Patterns of Care and Their Correlates. Soc. Sci. Med..

[30] Yu W. (2008). The psychological cost of market transition: Mental health disparities in reform-era China. Soc. Probl..

[31] Liu S., Griffiths S.M. (2011). From Economic Development to Public Health Improvement: China Faces Equity Challenges. Public Health.

[32] Liang D., Mays V.M., Hwang W.-C. (2018). Integrated Mental Health Services in China: Challenges and Planning for the Future. Health Policy Plan..

[33] Tse S., Ran M.-S., Huang Y., Zhu S. (2013). Mental Health Care Reforms in Asia: The Urgency of Now: Building a Recovery-Oriented, Community Mental Health Service in China. Psychiatr. Serv..

[34] Xiang Y.-T., Yu X., Sartorius N., Ungvari G.S., Chiu H.F.K. (2012). Mental health in China: Challenges and progress. Lancet.

[35] Ezoe S., Noda H., Akahane N., Sato O., Hama T., Miyata T., Terahara T., Fujishita M., Sakamoto H., Abe S.K. (2017). Trends in Policy on the Prevention and Control of Non-Communicable Diseases in Japan. Health Syst. Reform..

[36] Chung Y.-C., Park S., Roh S., Lee B., Lee Y., Rami F.Z., Li L., Shen J. (2021). Mental Health Services and Research and Development in South Korea. Taiwan. J. Psychiatry.

[37] Nishi D., Ishikawa H., Kawakami N. (2019). Prevalence of Mental Disorders and Mental Health Service Use in Japan. Psychiatry Clin. Neurosci..

[38] Wang L., Wang Z., Ma Q. (2019). The development and reform of public health in China from 1949 to 2019. Global. Health.

[39] Shinfuku N. (2016). A history of mental health care in Japan: International perspectives. Taiwan. J. Psychiatry.

[40] Sun Y., Bao Y., Ravindran A., Sun Y., Shi J., Lu L. (2020). Mental Health Challenges Raised by Rapid Socioeconomic Transformations in China: Lessons Learned and Prevention Strategies. Heart Mind.

[41] Lee S.-U., Park J.-I., Lee S., Oh I.-H., Choi J.-M., Oh C.-M. (2018). Changing trends in suicide rates in South Korea from 1993 to 2016: A descriptive study. BMJ Open.

